# Spikes in Retinal Bipolar Cells Phase-Lock to Visual Stimuli with Millisecond Precision

**DOI:** 10.1016/j.cub.2011.09.042

**Published:** 2011-11-22

**Authors:** Tom Baden, Federico Esposti, Anton Nikolaev, Leon Lagnado

**Affiliations:** 1 Medical Research Council Laboratory of Molecular Biology, Hills Road, Cambridge CB2 0QH, UK

## Abstract

**Background:**

The conversion of an analog stimulus into the digital form of spikes is a fundamental step in encoding sensory information. Here, we investigate this transformation in the visual system of fish by in vivo calcium imaging and electrophysiology of retinal bipolar cells, which have been assumed to be purely graded neurons.

**Results:**

Synapses of all major classes of retinal bipolar cell encode visual information by using a combination of spikes and graded signals. Spikes are triggered within the synaptic terminal and, although sparse, phase-lock to a stimulus with a jitter as low as 2–3 ms. Spikes in bipolar cells encode a visual stimulus less reliably than spikes in ganglion cells but with similar temporal precision. The spike-generating mechanism does not alter the temporal filtering of a stimulus compared with the generator potential. The amplitude of the graded component of the presynaptic calcium signal can vary in time, and small fluctuations in resting membrane potential alter spike frequency and even switch spiking on and off.

**Conclusions:**

In the retina of fish, the millisecond precision of spike coding begins in the synaptic terminal of bipolar cells. This neural compartment regulates the frequency of digital signals transmitted to the inner retina as well as the strength of graded signals.

## Graphical abstract

**Graphical Abstract:**

**Figure:**
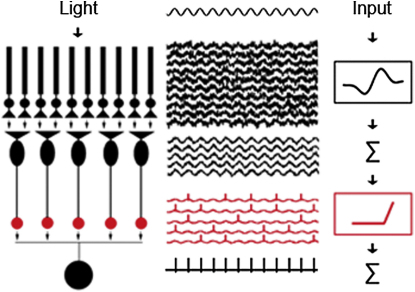

**Highlights:**

► The spike code of vision begins in retinal bipolar cells ► Spikes in bipolar cells phase-lock to visual stimuli with millisecond precision ► Spiking and graded calcium signals can switch on and off at individual synapses ► Spikes in bipolar cells encode a stimulus less reliably than spikes in ganglion cells

## Introduction

A key step in processing information from the outside world is the transformation of a sensory stimulus into the digital form of spikes. In the visual system, neurons can fire spikes with a precision of a few milliseconds relative to the timing of a stimulus [1–4], whereas in the auditory system precisions of hundreds of microseconds can be achieved. Such accurate timing of impulses allows information about the stimulus to be encoded not just in the average firing rate, but also in the time intervals between spikes [5]. A striking example of such a temporal code is found in fibers of the auditory nerve. These neurons convert the synaptic input from hair cells into spikes that are phase-locked to oscillations in sound pressure [6, 7].

In the visual system, signals with millisecond precision are found in the spike trains of retinal ganglion cells [2, 3, 8]. Where in the retina does the spike code originate? Photoreceptors generate graded changes in membrane potential in response to light, and it is widely thought that the visual signal remains in this analog form as it travels through bipolar cells after which it is converted into spikes by amacrine cells and ganglion cells in the inner retina [9–11]. However, a more complex picture of signaling in the retina has emerged recently. Imaging the presynaptic calcium transient in vivo has demonstrated that ON, OFF, transient, and sustained bipolar cells in zebrafish are all capable of generating calcium spikes modulated by visual stimulation [12]. This observation builds from electrophysiological studies demonstrating that spikes can be triggered by light in ON bipolar cells in slices of goldfish retina [13], as well as evidence that bipolar cells isolated from rats and fish contain sodium, calcium, and potassium conductances with the potential to support regenerative depolarizations and oscillations in membrane potential [13–20].

The idea that bipolar cells can encode a visual stimulus in the form of spikes as well as graded potentials raises fundamental questions about the function of the retinal circuit. What is the temporal precision of spikes in bipolar cells? How reliably do they encode a visual stimulus? And what is the relation between the visual signal encoded by graded signals and spikes? Here, we investigate these questions by using a combination of in vivo calcium imaging in zebrafish and electrophysiology in retinal slices from goldfish. The results in fish provide a new view of the functional plan of the retina in which the millisecond precision of digital coding begins with spikes generated in the synaptic compartment of bipolar cells.

## Results

### Presynaptic Calcium Transients in Bipolar Cells Exhibit Phase-Locking In Vivo

**Figure 1:**
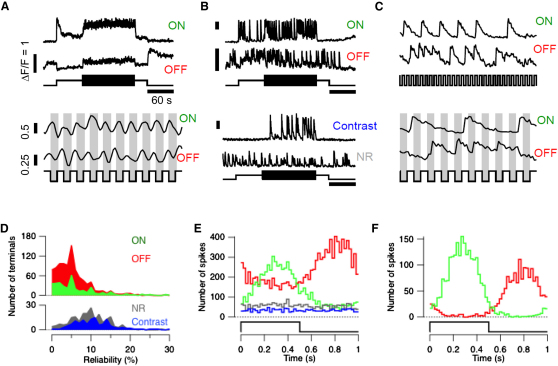
Phase-Locking of Calcium Spikes in the Synaptic Terminals of Bipolar Cells Observed In Vivo (A) Top: Examples of an ON and OFF terminal generating SyGCaMP2 responses continuously modulated by light. The stimulus consisted of a light step held at constant intensity for 60 s followed by modulation at 1 Hz and 100% contrast as a square wave for 120 s (lower trace). Bottom: Expansion of a 10 s segment of the upper records showing responses of the ON and OFF terminal in antiphase. (B) Top: Examples of an ON and OFF terminal generating spikes. Bottom: Examples of a terminal that did not generate a clear response to the onset or offset of steady light but responded to contrast, and a terminal that was nonresponsive (NR) to either aspect of the stimulus. The scale bars represent ΔF/F = 1, 60 s. (C) Expansion of spiking responses of the ON and OFF cell in (B). Note that both terminals became entrained to the stimulus for short periods at different multiples of the fundamental period. The lower panel shows that both ON and OFF terminals tended to be phase-locked to the stimulus, but with different phase-lags. The scale bars represent ΔF/F = 1. (D) Histograms showing the distribution of terminals with different reliabilities within each of the four classes shown in (B). (E) Timing of calcium spikes relative to the stimulus cycle for each of the four groups, color-coded as in (B and D). Note synchronization across terminals in the ON and OFF groups, but not in the contrast-sensitive or nonresponsive groups. (F) Timing of spikes in the 10% most reliable terminals in the ON and OFF group. The OFF cell was almost completely silent while the ON cell was active and vice versa. See also Figure S1.

To investigate how spikes in retinal bipolar cells transmit information about a visual stimulus, we began with an in vivo preparation—zebrafish expressing SyGCaMP2 under control of the ribeye A promoter [12, 21, 22]. SyGCaMP2 monitors presynaptic calcium transients that control neurotransmitter release. A full-field stimulus was modulated at 1 Hz (100% contrast, square wave) and Figure 1 summarizes results from 1821 terminals in five fish in which all strata of the inner plexiform layer (IPL) were sampled equally. Although some terminals generated purely graded responses to light (Figure 1A), 93% were also capable of generating fast calcium transients, probably reflecting voltage spikes in the presynaptic compartment (Figure 1B and Figure S1, available online).

Synaptic terminals were placed into four functional groups, and examples of each are shown in Figure 1B. The response to the onset or offset of steady light identified ON and OFF terminals, which constituted 21% and 52% of the total, respectively. The remaining terminals did not respond clearly to a light step, but 8% responded to contrast with spikes. Finally, 19% of terminals generated spikes that were not modulated by either stimulus. Bipolar cells generating strong, graded responses continuously followed the fluctuations in light intensity, with ON and OFF terminals in antiphase (Figure 1A, bottom). These graded calcium signals could be distinguished from spikes by their slower rate of rise, as illustrated in Figure S1. Notably, spikes occurred sparsely (Figures 1B and 1C), and the fraction of stimulus cycles in which a spike occurred (reliability) varied widely between different terminals, as shown by the histogram in Figure 1D. The mean reliability in the ON, OFF, and contrast-sensitive groups of terminals were 5.9%, 7.4%, and 11.4%, respectively.

Although presynaptic calcium spikes occurred sparsely, their timing was strongly dependent on the phase of the stimulus. For instance, in the ON cell in Figure 1C, most spikes were initiated while the luminance was high, whereas the OFF cell spiked while luminance was low. The tendency of a neuron to respond at a particular phase of a periodic stimulus is termed phase-locking, and in the auditory system this is one of the strategies by which neurons encode the frequency of sound [7]. The degree of phase-locking can be quantified as the vector strength [23], a metric that varies between zero (spikes independent of the stimulus) and 1 (perfect synchronization of all spikes at one particular phase-lag; Experimental Procedures). Vector strengths were indistinguishable in ON, OFF, and contrast-sensitive populations of terminals firing spikes, and averaged 0.5 ± 0.23. In the 10% of terminals with the highest reliability (averaging 19.9% ± 7.9%), the average vector strength increased to 0.75 ± 0.12 in ON terminals and 0.71 ± 0.12 in OFF terminals.

Phase-locking will cause a population of neurons to respond at a particular phase of a stimulus if those neurons tend to be synchronized. Such synchronization was found within the population of ON and OFF cells, as shown by the distribution of spike times relative to the stimulus cycle (Figure 1E). For instance, in the 10% most reliable terminals, the OFF group were almost completely silent at a phase of the stimulus in which the ON group spiked with the highest probability (Figure 1F). Synchrony within these bipolar cell populations probably arises from shared synaptic inputs received from photoreceptors. However, strikingly different behavior was observed in the 8% of terminals sensitive to contrast but of no clear polarity: spikes were distributed relatively uniformly as a function of phase-lag, indicating that shared photoreceptor input was not sufficient to synchronize activity within this population (Figure 1E).

### Light-Driven Spikes in Retinal Bipolar Cells: Electrophysiology

The visual systems of fish and mammals detect stimuli at frequencies of 20 Hz or more. What is the temporal precision with which spikes in bipolar cells encode these frequencies? This question was difficult to investigate by imaging SyGCaMP2 because the calcium transient detected by a high-affinity reporter decays with a time constant of about 1 s in the synaptic terminals of bipolar cells [12, 24]. To achieve the appropriate temporal resolution, we therefore turned to electrophysiological recordings in slices of goldfish retina.

**Figure 2:**
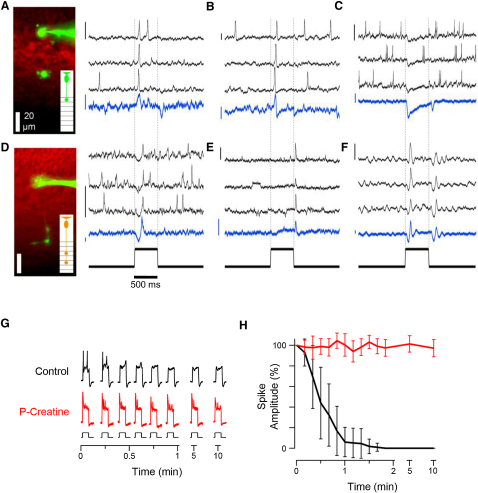
Light Modulates Spikes Recorded in Bipolar Cells in Retinal Slices (A–F) Examples of voltage spikes and oscillations recorded in the soma of six different bipolar cells with qualitatively distinct responses. In each panel, the black traces show three responses to the same 500 ms step of light (the scale bars represent 10 mV). The blue trace shows the average response to eight presentations of the stimulus where injection of a hyperpolarizing current, resulting in a voltage drop of −5 mV, prevented spike generation (the scale bars represent 2 mV). The currents used ranged between −2 and −20 pA in different cells. Examples of cells filled with Alexa 488 are shown in (A and D). The schematic insets illustrate the morphological classification of each cell (see also Figure S2). (A and B) are fast ON cells. (C) is a sustained OFF cell. (D) is a delayed ON cell. (E) is a transient OFF cell. (F) is a cell that generated damped oscillations at both light onset and offset. (G) Voltage responses of two bipolar cells to 500 ms steps of current injection, without (black) and with (red) creatine phosphate in the intracellular solution. (H) The average amplitude of a spike as a function of time after beginning whole-cell recording, normalized to the first (collected results from 44 cells). The error bars represent one standard error of the mean (S.E.M.). See also Figure S2.

Whole-cell recordings from a total of 128 bipolar cells were made with the pipette on the soma; 81% of the cells responded to current injection with spikes. A 500 ms step of light modulated spiking in 37 of 47 cells (79%), confirming that the population of bipolar cells made wide-spread use of spikes to encode a visual stimulus. Both graded and spiking responses occurred with high temporal precision, as shown by the six examples in Figures 2A–2F. For instance, in the ON cell in Figure 2A, spikes occurred at a mean interval of 1.6 s in the dark, but in 8/10 trials a single spike occurred 75 ± 9 ms after light onset. Injection of a small hyperpolarizing current to prevent the membrane potential crossing spike threshold revealed the underlying generator signal as a transient depolarization just after light onset and a transient hyperpolarization at light offset (blue trace). Other examples of cells generating spikes include a fast ON (Figure 2B), sustained OFF (Figure 2C), delayed ON (Figure 2D), and transient OFF (Figure 2E). Finally, Figure 2F shows a cell generating a damped voltage oscillation at both light onset and offset, similar to the electrical resonance reported in isolated bipolar cells [14]. The large majority of spikes in bipolar cells were generated by voltage-dependent calcium channels (L and/or T type), but some cells also fired sodium spikes blocked by TTX (Figure S2).

A number of studies have failed to clearly observe spikes in recordings made from bipolar cells in eyecup or slice preparations from a number of species [9, 10, 25]. Two factors may have made detection of spikes difficult in fish. First, recordings in the cell body do not faithfully detect spikes that originate in the synaptic terminal: the voltage signal detected in the soma is attenuated and filtered in time, at least to some degree, by the high resistance of the connecting axon and the capacitance of the soma (Figure S2). Second, the spike-generating mechanism could be destroyed by whole-cell dialysis. When recordings were made with a control intracellular solution, spikes were typically destroyed within 30–60 s (Figures 2G and 2H and Experimental Procedures). But when the solution in the pipette included 10 mM creatine phosphate, rundown could be prevented. Creatine phosphate is a mobilizable reserve of high-energy phosphates found in muscle and brain, commonly used to preserve the function of calcium channels, mitochondria, and synapses during electrophysiological recordings [26, 27]. It appears that spikes in bipolar cells are very sensitive to these energy stores, probably reflecting a regenerative mechanism dependent on voltage-sensitive calcium channels [14, 16].

In most neurons, spikes are generated in the axon initial segment, close to the soma. We could not, however, record spikes in any of 21 axotomized bipolar cells, as judged by dye-filling. In contrast, spikes occurred in 104 of 128 cells in which the terminal remained attached to the axon (Figures 2A and 2D). These results provide strong support for the idea that fast presynaptic calcium transients observed in vivo are caused by voltage spikes generated in the synaptic terminal (Figure 1 and [12]). Our observations are consistent with previous reports of spikes in an ON class of bipolar cell in the retina of goldfish [13, 16, 20], and further reveal that spikes are used to encode light in OFF cells and in neurons with varying kinetics, including those generating sustained signals.

### Spikes in Bipolar Cells Phase-Lock with Millisecond Precision

**Figure 3:**
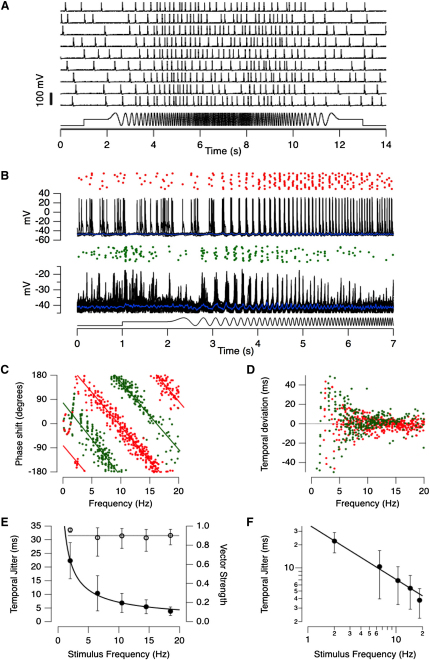
Temporal Precision of Spikes in Retinal Bipolar Cells (A) Nine responses of a bipolar cell to a stimulus in which light intensity was modulated as a sinusoid (100% contrast) and the frequency swept linearly with time from 0 to 20 Hz over a 5 s period and then back (bottom trace). Responses varied from trial-to-trial and the probability of spike generation is a function of frequency. (B) Comparison of responses in an OFF cell (top) and ON cell (bottom). Stimulus as in (A), but only the first half is shown. From top: raster-plot of spikes times, superimposed voltage responses (25 trials in the OFF cell and 11 trials in the ON), and averaged generator potential when hyperpolarized to prevent spiking (blue traces, −4 pA). The jitter in spike time is larger when light is modulated at lower frequencies. (C) The phase shift of each spike plotted as a function of stimulus frequency for the ON cell (green) and OFF cell (red) in (B). Phase shift was measured relative to the first zero crossing of the stimulus cycle in which the spike occurred. (D) The time deviation of each spike compared to a noise-free linear system responding with the same delay, represented by the line fits. (E) Standard deviation of spike times (jitter) as a function of stimulus frequency, averaged across bins of 4 Hz (filled circles). Corresponding values of vector strength are shown by the open symbols. (F) Temporal jitter as a function of stimulus frequency displayed as a log-log plot. The line fitting the points has a slope of −0.72. All error bars represent one S.E.M.

To explore the temporal precision with which spikes in bipolar cells encoded a visual stimulus, the intensity of full-field light was modulated sinusoidally at 100% contrast and the frequency swept up from 0 to 20 Hz, and then back down. This chirp stimulus was applied 10–50 times, and Figure 3A illustrates how the response of an individual cell varied from trial-to-trial. An expanded view of the response of an ON and OFF cell is shown in Figure 3B. In both channels, spike times were consistent between presentations, as can be appreciated by the raster plot (top), and the superimposition of voltage recordings (below).

Responses to the chirp stimulus were analyzed with the approach illustrated in Figures 3C–3E, which show ON and OFF cells as examples. First, the phase shift between each spike and the stimulus cycle in which it occurred was plotted as a function of frequency (Figure 3C). Phase shift varied linearly with frequency, as shown by the lines fitted to the points, indicating that the phase composition of the signal from both cells was preserved. As a result, ON and OFF responses remained in antiphase at frequencies up to 20 Hz (cf. Figures 1E and 1F). In other words, the mean time delay between the firing of the ON cell and OFF cell was maintained at half the stimulus period, independent of changes in frequency.

**Figure 4:**
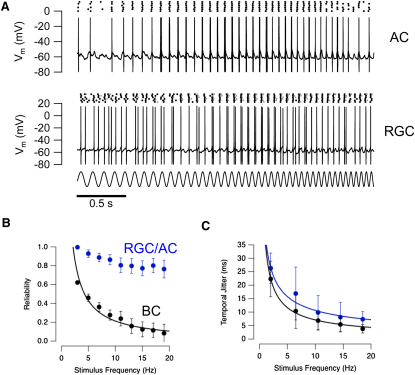
Spike Generation in Bipolar Cells Was Less Reliable than in Postsynaptic Amacrine Cells and Ganglion Cells (A) Voltage recording in an amacrine cell (top) and a retinal ganglion cell (bottom) in response to the same chirp stimulus used with bipolar cells. These are 3 s clips of single trials in which one or more spikes were generated in response to almost every cycle of the stimulus. The raster plots above shows spike timing in six trials. The first spikes are displayed in black, whereas follower spikes are displayed in gray. Spikes were generated with almost 100% reliability until the highest frequencies were reached. (B) Reliability of spike generation as a function frequency in bipolar cells (black; n = 8) compared with postsynaptic amacrine cells and ganglion cells (blue; n = 4 and 2, respectively). (C) Temporal jitter as a function of frequency calculated in the same set of experiments as in (B). Amacrine cells and ganglion cells are more reliable than bipolar cells at higher frequencies but do not display improved temporal precision (paired t test, 95% confidence). All error bars represent one S.E.M.

The next step in the analysis was to investigate how the temporal precision of spiking varied as a function of frequency. The time deviation of each spike from the line describing the response of the linear system best fitting the data was measured (Figure 3C). Figure 3D shows that the dispersion of this timing deviation fell as frequency increased for both the ON and OFF cell. Finally, the standard deviation in spike times over bandwidths of 4 Hz was calculated. This quantity, the temporal jitter, is plotted as a function of frequency in Figure 4E, with results averaged over eight cells (four ON and four OFF). Temporal jitter fell as frequency increased according to a power function, as shown by the log-log plot in Figure 3F (slope −0.72). Over the 16–20 Hz window, the temporal jitter was 2.2 ms in the OFF cell shown in Figure 3B and 2.8 ms in the ON cell, and averaged 3.8 ± 1.6 ms (n = 8). The strength of phase-locking was then quantified through the relation between vector strength and temporal jitter (Experimental Procedures). Vector strength was constant across frequencies at a value of 0.9 (Figure 3E), which is similar to that observed in auditory nerve fibers [6].

### Low Reliability of the Spike Code in Bipolar Cells

A key difference in the spike code of neurons in the inner retina emerged when we compared the reproducibility of their responses to repeated presentations of the chirp stimulus: spikes in amacrine cells and ganglion cells were generated much more reliably than spikes in bipolar cells. Example records in an amacrine cell and ganglion cell are shown in Figure 4A, in which each cycle of the chirp elicits a single or double spike with a probability approaching 100%. In contrast, when the response to this stimulus was recorded in a bipolar cell, no single cycle of the chirp stimulus consistently elicited a spike, as can be seen in the examples in Figure 3. To quantify the reliability of signaling, we counted the fraction of stimulus presentations in which a given cycle of the stimulus generated one or more spikes. Across a sample of eight bipolar cells reliability fell as a function of frequency and averaged ∼15% above 15 Hz (Figure 4B, black). In contrast, the reliability of postsynaptic amacrine cells and ganglion cell did not fall below 80% (blue). Despite these differences in reliability, the temporal precision of spikes were similar across all three types of neuron (Figure 4C).

### Spike Generation Did Not Significantly Alter Frequency Tuning

Temporal filtering of the visual signal can be shaped by active conductances within neurons [28–30]. For instance, comparison of the generator potential with the spike output demonstrates that the spike-generating mechanism acts as a high-pass filter in cat retinal ganglion cells [31] and as a band-pass filter in ocellar neurons in the cockroach visual system [32]. In the case of bipolar cells, interactions between voltage-sensitive calcium channels and calcium-activated potassium channels in the synaptic terminal can generate both spikes and an electrical resonance [14, 20]. Does the spike-generating mechanism in bipolar cells alter the time course of the visual signal?

**Figure 5:**
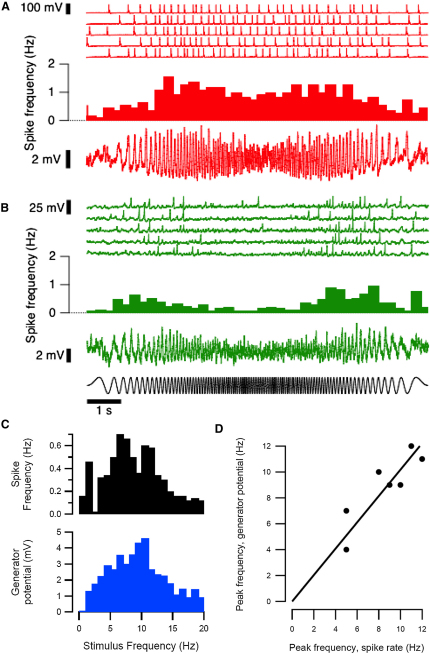
Spikes and Graded Responses Represent Similar Temporal Filters (A) Comparison of the temporal filters describing the generation of spikes and graded changes in membrane potential in an OFF cell. The stimulus is shown at the bottom of (B). Top: Voltage responses in five of the 25 trials. Middle: Histogram showing the mean spike rate over different time bins. Bottom: Graded generator potential recorded while injecting hyperpolarizing current to prevent spike generation (−4 pA, average of 10 trials). (B) The same measurements as in (A) made in an ON cell. (C) Spike rate (top) and amplitude of the generator potential (bottom) as a function of frequency for the OFF cell in A. (D) The relation between the peak frequency for transmission of the generator potential and spikes. Each point represents one cell. The line has a slope that is not significantly different from equality (1.02 ± 0.06).

To investigate the transfer function of bipolar cells, the retina was stimulated 10–50 times with a chirp. In the OFF cell in Figure 5A, the probability of firing peaked at a stimulus frequency of 11–12 Hz, and when spiking was prevented by injection of hyperpolarizing current, the graded voltage signal also exhibited bandpass tuning with maximum amplitude at the same frequency (Figure 5C). The ON cell in Figure 5B also exhibited bandpass behavior at the level of both spikes and the underlying generator potential, but with peak gain at 7–8 Hz. The relation between the peak frequency for transmission of the generator potential and spikes is plotted for each of seven cells in Figure 5E. The line through the points has a slope which is not significantly different from equality (1.02 ± 0.06). We conclude that the spike-generating mechanism in bipolar cells does not significantly affect frequency tuning but simply converts an analog (graded) signal to a digital (spiking) format.

### Switches in the Signaling Mode of Individual Terminals

**Figure 6:**
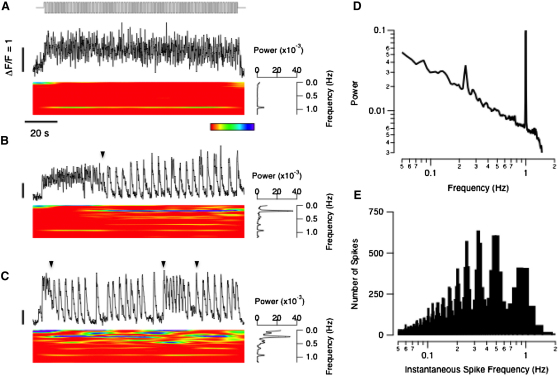
Calcium Spikes and Graded Calcium Signals: Switches in Signaling Mode (A) Spectral and time-frequency analysis of a terminal that did not generate spikes. The stimulus is shown at the top (square wave, 100% contrast, 1 Hz). The time-frequency plot is below, and the blue end of the spectrum represents higher powers, and to the right is the power spectrum averaged over the period of light modulation. The power of the response is continuously concentrated at a frequency of 1 Hz. (B) Example of a terminal that began signaling light modulation in a purely graded manner, but then switched to signaling predominantly through spikes (arrowhead). The power was concentrated at 1 Hz and 0.25 Hz. (C) Example of a terminal in which spiking switched between different frequencies, predominantly 0.25, 0.5, and 1 Hz (arrowheads). The power is more evenly distributed between 0 and 1 Hz, and the terminal switched between 1/4, 1/2 and 1 Hz. The scale bars represent ΔF/F = 1. (D) Average power spectrum of the SyGCaMP2 signal across all 1821 terminals, irrespective of whether they generated spikes. Dominant frequencies were 1/4 and 1 Hz. (E) Spectrum of spiking signals illustrated in the form of a histogram showing the distribution of instantaneous spike frequencies (i.e., the reciprocal of the interspike interval). More spikes were centered around 1/4, 1/3, and 1/2 Hz compared to 1 Hz.

To investigate how graded and spiking signaling modes interact in the synaptic terminal of bipolar cells, we analyzed SyGCaMP2 signals in the time-frequency domain with a Wigner Distribution Function [33]. Of 1818 terminals imaged in vivo, only 39 generated a purely graded signal that followed a stimulus modulated at 1 Hz (100% contrast), and an example is shown in Figure 6A. The accompanying spectrogram confirms that the modulation in presynaptic calcium was locked on to the 1 Hz stimulus, as can also be seen in the power spectrum. However, the vast majority of terminals (1400) generated spikes as well as a continuous presynaptic calcium signal, and an example is shown in Figure 6B. Although this terminal began responding to the stimulus in a graded manner (i.e., simply following the 1 Hz stimulus), after about 30 cycles it also began to spike at 0.25 Hz. The next transition involved a complete switch in signaling modes: the continuous component of the presynaptic calcium signal stopped and spikes at 0.25 Hz took over. As a result, the power spectrum averaged over two minutes of the periodic stimulus contained peaks at 1 Hz and at 0.25 Hz, although with weaker components at 1/2 and 1/3 the fundamental frequency also evident.

A second example of a terminal switching between different signaling states is shown in Figure 6C. The amplitude of the signal at 1 Hz waxed and waned, but the spiking component contained more power and switched between frequencies of 1/2, 1/3, 1/4, and 1/5 Hz. All spiking terminals demonstrated sudden changes in frequency over the period of stimulation, and these were invariably accompanied by modulation of the graded signal. Apart from rapid adaptation of the initial response to the stimulus, these changes in spike frequency occurred randomly: we could not detect time-dependent trends over the whole population of 1769 spiking terminals. In 35% of terminals, the continuous calcium signal disappeared almost completely for some periods.

To assess the relative contributions of graded and spiking responses across the population of bipolar cells we averaged the power spectra of SyGCaMP2 signals from all 1821 terminals, regardless of response (Figure 6D). The two dominant modes of encoding the stimulus were continuous modulations of calcium at 1 Hz and phase-locking of spikes at four times the fundamental period. To isolate the contribution of spikes, we measured interspike intervals across the whole sample of cells and plotted the distribution of instantaneous spike frequencies (Figure 6E). In this case, the dominant signal was not at 1 Hz: more spikes fell around the peaks corresponding to frequencies of 1/2, 1/3, and 1/4 Hz.

Three aspects of the behavior shown in Figure 6 are notable. First, graded and spiking signaling modes often occurred simultaneously in an individual terminal. Second, spiking responses could jump up and down in frequency, and even switch on and off. Third, the amplitude of the graded component of the presynaptic calcium signal could be strongly modulated.

### A Narrow Voltage Window for Spike Generation

**Figure 7:**
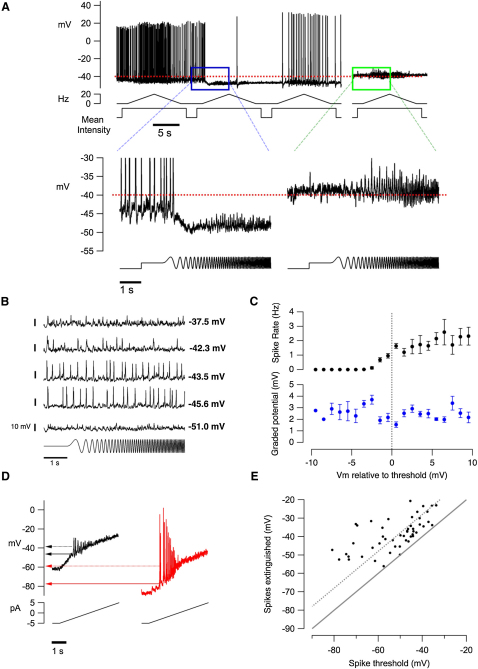
Spiking Is Switched on and off by Fluctuations in Resting Membrane Potential (A) An example of spontaneous changes in the steady (resting) membrane potential in the soma of a bipolar cell recorded in a slice. The dashed red line indicates the threshold for spiking, at −40 mV. The blue box highlights a sudden hyperpolarization in the resting potential below the threshold that switches off spiking in response to the frequency sweep. This part of the record is expanded below. As the resting potential gradually increases, spiking is switched back on, but at the lower resting potential the stimulus triggers spikes less effectively. The green box shows a later period in the same recording, where the resting potential became depolarized above threshold. Note that spikes are extinguished, but the graded generator signal is not; this is shown more clearly in the expanded trace below. (B) An example of imposed changes in the resting membrane potential that switch spiking on and off. At −51 mV, no spikes were evident (I_hold_ = −8 pA), but they were apparent at −45.6 mV (I_hold_ = −2 pA). At −42.3 mV (I_hold_ = +2 pA), spikes were lower in amplitude, and at −37.5 mV (I_hold_ = +10 pA) they were extinguished. (C) Mean spike rate (above) and mean generator potential (below) as a function of the resting membrane potential relative to threshold. Collected results from the protocol shown in (B) (238 trials in eight cells). Mean spike rate and generator potentials were measured at each cell's best frequency (±4 Hz). The error bars represent one S.E.M. (D) Example of voltage responses of two bipolar cells in response to ramp current injection. This protocol was used to determine the spike threshold (solid arrows) and the potential at which spikes were extinguished (dashed arrows). (E) The potential at which spikes were extinguished plotted as a function of spike threshold. Collected results from 49 cells. The solid line signifies equality and the dashed line is offset by +12 mV; the majority of cells generated spikes within this voltage window. The voltage window was 10 mV or less at resting potentials above −46 mV.

To investigate the mechanisms that might underlie spontaneous changes in the efficiency of spike generation, we used retinal slices to make prolonged voltage recordings in bipolar cells while stimulating with repeated chirp stimuli, as shown by the example in Figure 7A. In this cell the resting potential at the start of the recording was −43 mV while the spike threshold was −40 mV (indicated with the dashed red line), allowing a generator potential of only a few mV to drive firing. Spikes were suddenly switched off by a spontaneous hyperpolarization of 5 mV that prevented the voltage from reaching threshold (indicated by the blue box). Subsequently, a slow depolarizing drift in membrane potential led to restoration of the spiking response to light but with lower efficiency.

Spiking could also be blocked by depolarization of the resting potential above the spike threshold. In the example in Figure 7A, a depolarization to −38 mV was sufficient to block all light-driven spikes, as shown by the later period of recording from this cell boxed in green. Drifts in resting potential of ±5 mV were observed in all 121 cells in which recordings were maintained for periods of minutes, causing the efficiency of spike generation to vary continuously. In 10% of cells, sudden flips in membrane potential were also observed.

To test systematically how membrane voltage affected spike generation we held bipolar cells at different potentials by injection of small currents from the cell body and measured voltage responses to light. In the example in Figure 7B, spike threshold was −46 mV, and spiking was extinguished at −38 mV. Population data from 238 stimulus presentations in eight cells are shown in Figure 7C. The transition from purely graded signaling to a combination of spikes and graded signals occurred over a voltage range of less than ∼3 mV. The width of this window probably reflects the amplitude of the graded voltage signal, which also averaged 4 mV (lower graph). A second method for determining the voltage window for spike generation was to apply a current ramp. Two examples are shown in Figure 7D: a cell generating small slow spikes from a threshold of −45 mV (probably driven by L-type calcium channels) is shown in black, and a cell generating fast large spikes from a threshold of −78 mV (probably driven by sodium channels) is shown in red. Collected results from 49 bipolar cells are shown in Figure 7E, which plots the relation between the spike threshold and the potential at which spikes were extinguished. In none of these cells could spikes be observed at a potential above −35 mV, and in the majority the voltage window for spike generation was less than 12 mV.

Together, the results in Figure 7 demonstrate that the reliability of spike generation in the synaptic terminal of bipolar cells is finely controlled by small changes in membrane potential. This behavior provides an explanation for mode-switching imaged in vivo: spikes can be switched off by small and spontaneous hyperpolarizations that prevent the membrane potential from crossing threshold or by small depolarizations that extinguish the spike-generating mechanism. These results also provide an explanation for changes in the amplitude of the graded calcium signal observed in vivo (Figures 6B and 6C): the threshold for activation of the L-type calcium current is around −43 mV [34], and in many bipolar cells the resting membrane potential fluctuated around this value (Figures 6B and 6C).

## Discussion

The classical picture of signal flow in the retina categorizes bipolar cells as simply graded neurons in which the visual signal continuously modulates the membrane potential, but this study demonstrates that bipolar cells also transmit the visual signal to the inner retina by using spikes that phase-lock to a visual stimulus with millisecond precision. In the future, it will be important to understand whether spikes in retinal bipolar cells occur in species other than fish.

### Sensory Signaling across Ribbon Synapses

Of all our senses, the two operating on the fastest timescales are hearing and vision, and there are some striking parallels in the machinery used to transmit the first auditory and visual signals. The synaptic output of hair cells drive the first spikes in the auditory system with a precision of hundreds of microseconds, allowing phase-locking to sounds at kHz frequencies, and here we have demonstrated that the first spikes in the visual system display a similar degree of phase-locking at frequencies relevant to vision. Like photoreceptors and bipolar cells, cochlear hair cells possess a special ribbon structure holding glutamatergic vesicles close to the active zone, and these vesicles can be released very rapidly and efficiently. It appears, therefore, that ribbon synapses have evolved to reliably transmit fast sensory signals. But ribbon synapses have another outstanding property—they are also capable of transmitting graded voltage signals by modulating a slower but continuous mode of neurotransmitter release [35]. The coexistence of strong pulse-like neurotransmission with a slower continuous vesicle cycle can now be understood in the light of the present study: ribbon synapses of bipolar cells transmit both analog and digital signals.

### What Is the Relative Importance of Analog and Digital Signals in Bipolar Cells?

We now need to understand how analog and digital signals transmitted by bipolar cells affect the spike code that ganglion cells transmit to the brain. Factors to consider include the relative efficiencies with which graded and spiking signals drive glutamate release, the relative frequencies of these signals at individual synapses, and the integration of these different inputs by convergence onto postsynaptic amacrine cells and ganglion cells.

A number of observations indicate that spikes will drive vesicle fusion at higher rates than graded signals. Presynaptic calcium transients triggered by spikes were faster than graded calcium changes, indicating that they were driven by larger calcium currents (Figures 1 and 2 and Figure S1). Capacitance measurements in mixed bipolar cells of goldfish also demonstrate that a single calcium spike can release the whole of the readily releasable pool of vesicles (RRP) [16]. The RRP contains ∼20 vesicles per active zone that can be released with a rate constant of 500 s^−1^ at −10 mV, but only 25 s^−1^ at −35 mV [34]. The RRP is also refilled rapidly after a spike [36, 37], so that about eight vesicles can be released per spike per active zone at firing rates of 5 Hz [16].

Although spikes are expected to trigger vesicle release at higher instantaneous rates than graded signals, they were infrequent. Bipolar cells rarely fired faster than a few Hz (Figures 1–3), causing faster stimuli to be undersampled (Figure 4C), whereas graded signals occurred continuously for prolonged periods. Understanding the relative importance of the graded and spiking signaling modes will require direct comparisons of how they drive neurotransmission from individual synaptic terminals.

Imaging presynaptic calcium indicated that the efficiency with which graded signals are transmitted will vary. For instance, Figures 6B and 6C show periods when the generator potential made no significant contribution to the calcium signal in the terminal, which was dominated by fast spikes. Modulation of the graded SyGCaMP2 signal was sometimes profound enough to turn it off completely (Figure 6). GCaMP2 has an apparent K_d_ of ∼150 nM, and it is known that submicromolar levels of calcium are sufficient to drive continuous release [12] so these results suggest that graded transmission of the visual signal to the inner retina can be switched on and off.

### Changes in the Reliability of the Spike Code in the Inner Retina

Spikes in amacrine cells and ganglion cells encoded visual stimuli much more reliably than spikes in bipolar cells (Figure 4B). How is this dramatic improvement in reliability achieved? One mechanism is likely to be convergence of many bipolar cell inputs onto the postsynaptic neurons. Convergence is a fundamental feature of the retina's design, aiding the extraction of signals by causing uncorrelated noise sources to cancel [11]. The number of inputs from bipolar cells varies between RGCs with dendritic trees of different sizes. In zebrafish, the average convergence factor is ∼7:1, and can be as low as 1:1 in the fovea of primates. In the cat, an alpha ganglion cell can collect ∼1000 synaptic contacts from ∼140 bipolar cells [38]. Assuming that spikes in bipolar cells are more effective in triggering vesicle release compared to graded potentials, a design in which an RGC receives many spiking inputs from bipolar cells would have the potential advantage of making the spike train leaving the RGC less susceptible to noise within that one neuron. This idea echoes the observation that variability in the timing of spikes in ganglion cells reflects temporal variability in synaptic inputs rather than noise intrinsic to the ganglion cell [8]. Of course, if reliable spike generation in an RGC requires integration of many spatially distributed and sparsely active inputs, this will come at the cost of reduced spatial resolution.

### The Synaptic Compartment of Bipolar Cells as a Computational Unit

Imaging presynaptic calcium in vivo demonstrated that the transformation of graded signals into spikes could change over time, and individual bipolar cells switch the emphasis between these two modes of signaling (Figures 1, 6, and 7). Modulation of the spike-generating process is not unexpected because the synaptic terminal of bipolar cells is a key site of integration: synaptic currents originating from photoreceptor inputs in the dendrites combine with inhibitory synaptic inputs received directly from amacrine cells [11, 39]. Over longer timescales, the electrical properties of the terminal can be altered by a number of neuromodulators released under different conditions of adaptation, including dopamine, somatostatin and endocannaninoids [40–42]. Activation of ionotropic and metabotropic receptors on the terminal membrane can be expected to regulate spike generation by altering the voltage-dependence of calcium channel activation, the threshold for regenerative depolarization, and the properties of voltage noise [8, 43]. Indeed, spikes were elicited only within a narrow voltage range, which could be exceeded by spontaneous changes in membrane voltage recorded in a slice (Figure 7).

It will be interesting to investigate how far adaptation within the retinal circuit involves modulation of the conductances controlling the conversion of graded signals into spikes in the synaptic terminal of bipolar cells.

## Experimental Procedures

### Multiphoton Imaging in Zebrafish In Vivo

All procedures were carried out according to the UK Animals (Scientific Procedures) Act 1986 and approved by the UK Home Office. Transgenic zebrafish expressing SyGCaMP2.0 were imaged at 9–11 dpf as described previously [21]. The multiphoton microscope was controlled with ScanImage v.3.6 software [44], and movies were processed with the SARFIA suite of analysis routines [22] running in Igor Pro 6 (Wavemetrics). These routines begin with the automated extraction of the fluorescence change in each terminal by defining regions of interest with a filtering algorithm based on a Laplace operator.

Light stimuli were delivered with an amber light-emitting diode (LED) (590 nm) filtered through a 600/10 BP filter and projected through a light guide onto the surface of the bath, close to eye of the fish. The mean intensity of light stimuli was ∼2 × 10^5^ photons/μm^2^/s, which corresponds to a low photopic intensity.

### Electrophysiology in Retinal Slices

Adult goldfish were dark adapted for 30 min and killed by decapitation followed by pithing. Further procedures were performed under infrared illumination. Eyes were enucleated and retinas placed in oxygenated AMES medium (255 Osm, 2.1 mM CaCl_2_) containing 5% hyaluronidase for 2 min to digest the vitreous humor. The remainder of the vitreous was removed with forceps. A small part of the retina was placed on filter paper (0.45 μm; Millipore), sliced into 200 μm strips and mounted in the recording chamber with stripes of Vaseline for immediate electrophysiological recordings at room temperature (21°C) with methods described previously [45]. The remaining tissue was placed in oxygenated AMES solution at 4°C for later use.

Slices were viewed under an Olympus BX51WI upright microscope with a 60× water immersion objective (N.A. 0.9) with oblique IR illumination and an EM-CCD Camera (C9100-13, Hamamatsu). Cells were visualized by filling with Alexa-488 and taking images taking through a standard GFP filter set. Electrodes of 7–9 MΩ resistance were filled with (in mM): 104 Kgluconate, 8 KCl, 2 MgCl_2_, 4 HEPES, 0.5 EGTA, 10 disodium phosphocreatine (Sigma), 2 MgATP, 1 NaGTP, 1 NacGMP, and 0.05 Alexa 488 (240 mOsm, pH: 7.4). The calculated junction potential was −12 mV. The control intracellular solution used in Figure 2G was identical, except that phosphocreatine was replaced with equimolar potassium gluconate. Signals were recorded with an Axopatch 200B amplifier (Molecular Devices), interfaced with an ITC-16 (HEKA), and controlled with Pulse Control 4.3 running under Igor Pro 5 (Wavemetrics). Unless stated otherwise, the holding current in the current clamp configuration was 0 pA. Light stimuli were delivered through the objective with a blue LED. The intensity of the stimulus was modulated by switching the LED on and off at 10 kHz and varying the duty cycle. The mean intensity was adjusted to ∼2 × 10^5^ photons/μm^2^/s.

### Analysis

Records obtained by imaging and electrophysiology were analyzed with Igor Pro 6 (Wavemetrics). To detect calcium spikes in SyGCaMP2 recordings, we used a procedure that is illustrated in Figure S1 and which is a modified version of that used by [12]. Individual records were normalized as the relative change in fluorescence (ΔF/F), smoothed by binomial Gaussian filtering, and then differentiated. The resulting trace was then compared with a threshold set at ∼1.8 times the standard deviation over a single recording period (typically a few minutes). The reliability of the algorithm was tested by comparing the results with manual spike detection.

Time-frequency analysis of SyGCaMP2 signals was performed with a Wigner Distribution Function (WDF). The choice of the WDF approach over alternatives such as Wavelets was motivated by the proximity of the Nyquist frequency (3.6 Hz) to the frequency band of interest.

To detect spikes in voltage recordings, we used a procedure similar to that used to detect spikes in SyGCaMP2 signals: the trace was differentiated and upward crossings through an appropriate threshold detected. The degree of phase-locking was quantified as vector strength with the method introduced by [23]:

$$vector\;strength=\frac{1}{n}\sqrt{\sum_{i}^{n}{\left(\cos\left(\frac{2\pi t_{i}}{T}\right)\right)}^{2}+{\sum_{i}^{n}\left(\sin\left(\frac{2\pi t_{i}}{T}\right)\right)}^{2}}$$

where *t_i_* is the time interval between the beginning of a stimulus cycle and the *i*th spike, *n* is the total number of spikes, and *T* is the stimulus period. The vector strength, *r,* is related to the temporal jitter [6]:

$$s=\frac{\sqrt{2\left(1-r\right)}}{2\pi f}$$

where *s* is the temporal jitter and *f* the frequency.

Unless stated otherwise, errors are one standard deviation.

## Supplemental Information

Document S1. Supplemental Experimental Procedures and Three Figures
