# Supplementary material for: Spikes in Retinal Bipolar Cells Phase-Lock to Visual Stimuli with Millisecond Precision
Source: Curr Biol. 2011 Nov 22;21(22):1859–69. doi: 10.1016/j.cub.2011.09.042 (PMC3235547; doi:10.1016/j.cub.2011.09.042)
Supplement: Document S1. Supplemental Experimental Procedures and Three Figures [file mmc1.pdf]

Current Biology, Volume *21*

Supplemental Information

Spikes in Retinal Bipolar Cells

Phase-Lock to Visual Stimuli

with Millisecond Precision

Tom Baden, Federico Esposti, Anton Nikolaev, and Leon Lagnado

Supplemental Inventory

1. Supplemental Experimental Procedures: Algorithm for automatic spike detection
2. A variety of spike-generating mechanisms in different types of bipolar cell.
Related to Figure 2.
3. The axon attenuates the transfer of spikes from the synaptic compartment to
soma. Related to Figure 2.
4. Supplementary References.

Supplementary Information

Spikes in retinal bipolar cells phase-lock to visual stimuli with millisecond precision

Tom Baden, Federico Esposti, Anton Nikolaev and Leon Lagnado

MRC Laboratory of Molecular Biology, Hills Road, Cambridge CB2 0QH, UK

1. Supplemental Experimental Procedures: Algorithm for automatic spike detection

To detect calcium spikes in the SyGCaMP2 recordings we used a procedure that is illustrated in Supplementary Figure 1[1]. To illustrate the ability of the algorithm to differentiate between graded and spiking signals we use an example trace that contains both forms of signal (Fig. S1A). Individual records were normalized as the relative change in fluorescence (ΔF/F), smoothed by binomial Gaussian filtering (Fig. S1B), and then differentiated (Fig. S1C). The resulting trace was then compared with a threshold (red line in Fig. S1C) set at ~1.8 times the standard deviation measured over a single recording period (typically a few minutes). Fig. S1D shows the spikes detected in this record. The reliability of the algorithm was tested by comparing the results with manual spike detection over
many traces.

Supplementary Figure 1

2. A variety of spike-generating mechanisms in different types of bipolar cell

The voltage signals recorded in the cell bodies of bipolar cells varied in amplitude, kinetics and frequency. Measured in the soma, spike amplitudes ranged between 11 and 85 mV (mean: 30 ± 24 mV, n = 138) and spike half-widths ranged between 3.0 and 30.3 ms (Mean: 11.5 ± 9.7 ms, n = 138). Eight individual examples are shown in

Supplementary Figure 3A, all in response to a “chirp stimulus”. In some cells, spikes measured at the soma were relatively fast and large (>25 mV; 4, 5, 6, 7), but in others they were smaller and less easily distinguished from slower graded signals (1, 2, 3). A minority of cells only generated small graded voltage signals without spikes (8).

Supplementary Figure 2

Application of 1μM TTX blocked large spikes in all 7 cells tested, demonstrating that these were driven predominately by TTX-sensitive Na^+ channels. These were all cone-driven bipolar cells. An example of the effects of TTX is shown by the black trace in Supplementary Figure 3B. The top panel shows the current response of an ON cell stepped from -60 to -20 mV while blocking K^+ currents with a Cs^+-based intracellular solution and 20 μM extracellular TEA: the transient inward current was completely abolished by 1 μM TTX. The lower panel shows large spikes recorded in the current-clamp configuration in response to a current ramp; again these were completely abolished by addition of 1 μM TTX. The effects of TTX were reversible.

Smaller amplitude spikes (<25 mV at the soma) were driven by Ca^{2+} channels. The lower panel in Supplementary Figure 3C shows an example in which such spikes were blocked by inhibiting L-type Ca^{2+} channels with 10 μM nifedipine (n= 64). Notably, many of these cells also contained Na^+ channels sensitive to TTX (Supplementary Figure 3C, top), and in these cases spikes were prevented by inhibiting either the Na^+ or Ca^{2+} conductance. Finally spiking in some classes of bipolar cell spikes was predominately driven by T-type Ca^{2+} channels that could be blocked with 10 μM NNC 55-0396 (n = 25, Supplementary Figure 3D). In these cells spiking was elicited by step depolarizations, but not when slowly ramping membrane potential towards spike threshold (Supplementary Figure 5D, bottom), consistent with gradual inactivation of T-Type Ca^{2+} channels during slow depolarization. The effects of calcium channel blockers were reversible.

The presence of L- and T- type Ca^{2+} channels as well as TTX-sensitive Na^+ channels in bipolar cells is consistent with previous reports using fish, salamander, rat, mouse and human [2-8]. It therefore seems that different combinations of

voltage-sensitive conductance generate spikes varying in speed, amplitude and adaptive properties in different types of bipolar cell.

3. The axon attenuates the transfer of spikes from the synaptic compartment to soma

The synaptic terminal of most bipolar cells is separated from the soma by a long thin axon (e.g Fig. 2B). As a consequence, a spike generated in the terminal is attenuated when recorded in the soma. To estimate the degree of spike attenuation in somatic recordings we constructed a simple model of bipolar cell passive membrane properties using NEURON (version 7.1). In all simulations, the soma was an ellipse with major and minor axese of 8 µm and 5 µm, while the terminal was a circle with a radius of 3 µm. The membrane resistance was 160 Ω/cm^2; capacitance 1 pF/cm^2 and leak conductance 71.4 $\mu S/cm^2$. Only the diameter and length of the axon were varied, and Fig. S2 shows the results of simulations using the averaged waveforms of Na^+ and Ca^{2+} spikes as the input in the terminal (black traces). The attenuated waveforms reaching the soma after filtering through axons of different length and diameter are shown by the colored traces. Depending on the waveform of the spike and on axon geometry, amplitude attenuation was typically between 30 and 70 %, and exceeded 90 % in extreme cases. Due to their faster kinetics, Na^+ spikes were attenuated more than Ca^{2+} spikes. In real recording situations, a spike recorded in the soma will also be attenuated by the access resistance of the recording pipette.

Supplementary Figure 3

Supplementary Figure 1

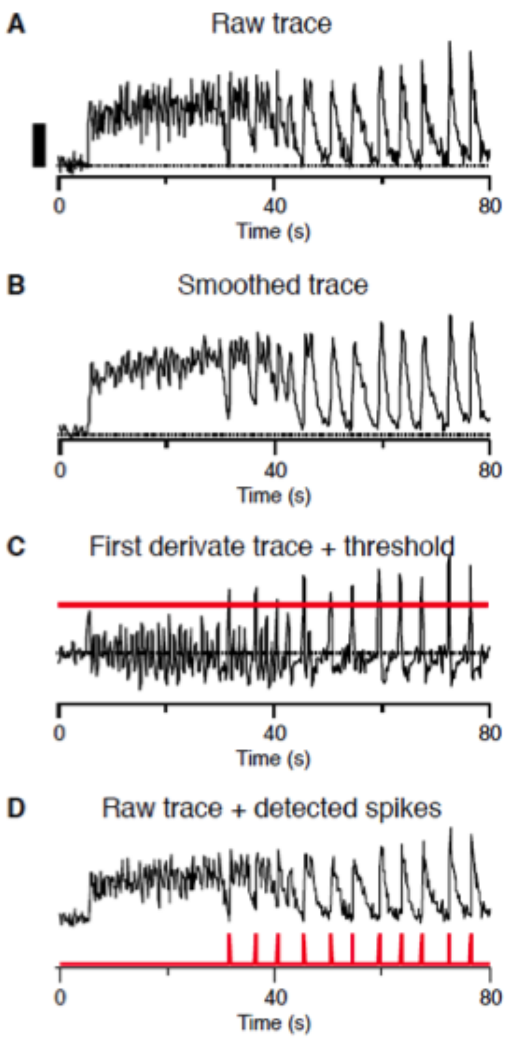

Supplementary Figure 2

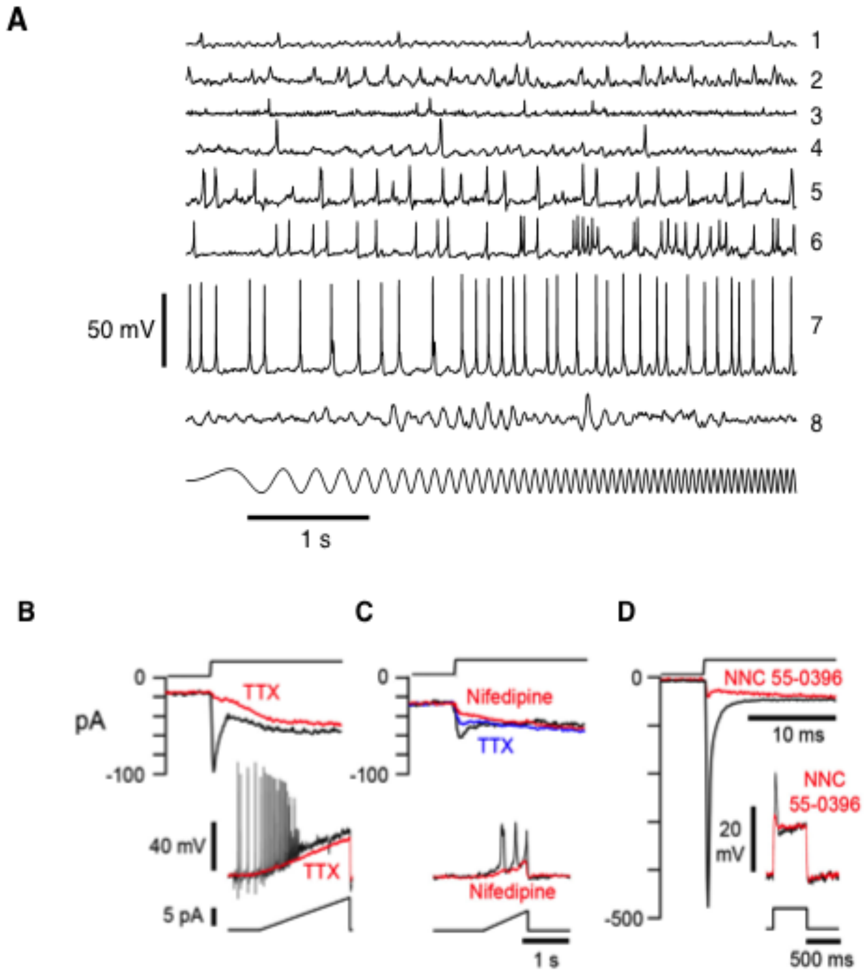

Supplementary Figure 3

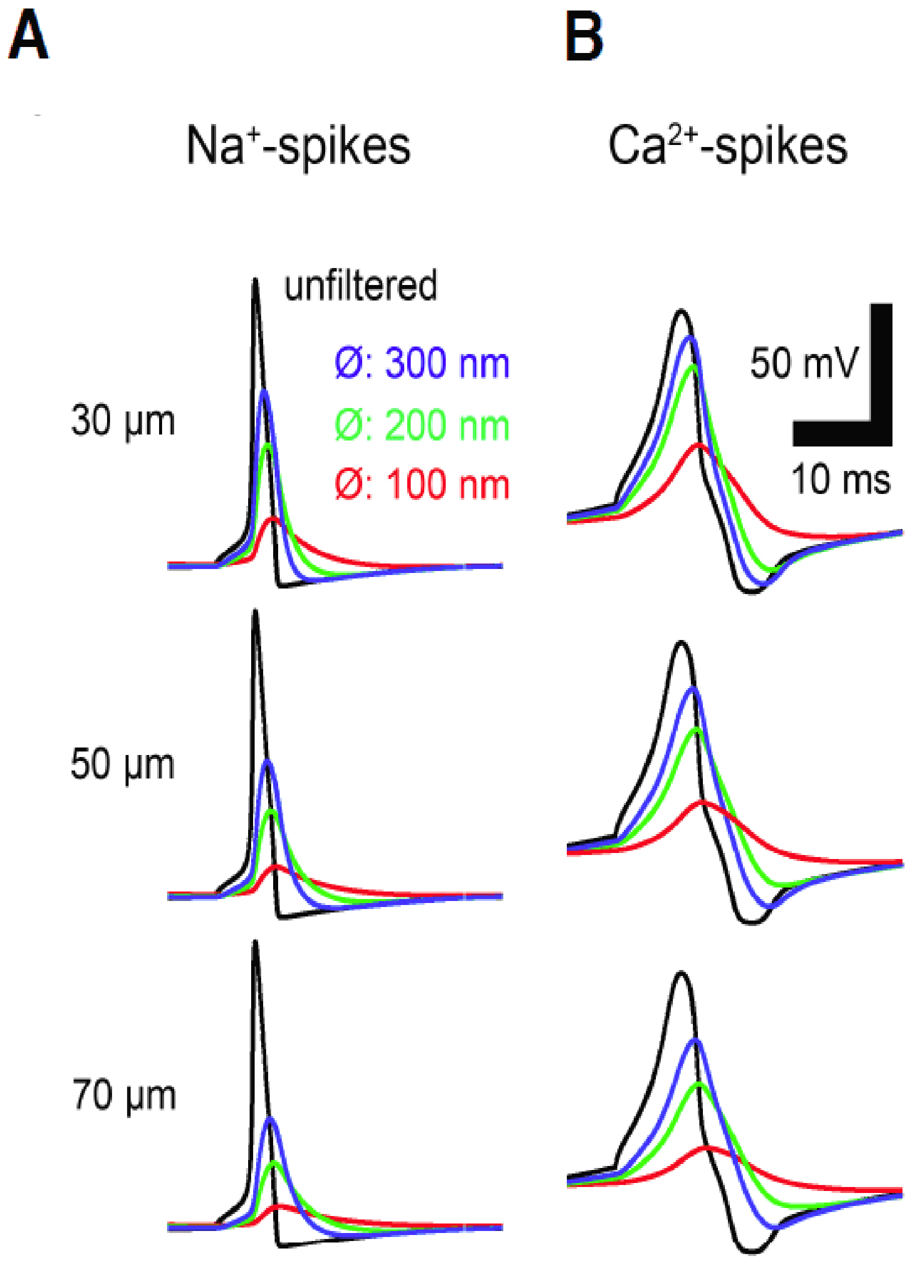

Supplementary Figure Legends

Figure S1, related to Figure 1. Algorithm for automatic spike detection

A. Raw SyGcaMP2 trace. Scale bar: DF/F = 1. **B.** Smoothed version of the original trace. A binomial Gaussian filter was used of order 7. **C.** Derivative of the smoothed trace. The red line shows the threshold for spike detection, which was set as 1.8 times the standard deviation of this same trace. **D**. Spike series in red compared with the original trace.

Figure S2, related to Figure 2. A variety of spike-generating mechanisms in different types of bipolar cell.

A. Eight examples of responses to a "chirp" stimulus in which light intensity was modulated as a sinusoid (100% contrast) and the frequency swept linearly with time from 0 to 20 Hz over a 5 s period. Note the variety of spike amplitudes, kinetics and frequencies recorded in the cell body. Cell 8 does not fire spikes but shows relatively large modulations in membrane potential that follow the stimulus. **B.** Sodium currents: the Na^+ channel blocker TTX (1 µM, red) abolished a transient inward current elicited by a voltage step from -60 to -20 mV (top) and abolished spikes in response to a 2 s depolarizing current ramp in another bipolar cell (bottom). **C.** L-type calcium current: (top) the $_LCa^{2+}$ channel blocker nifedipine abolished the remainder of a transient inward current (red, 10 µM) following bath application of TTX (blue), and abolished spikes in response to a depolarizing current ramp in another cell (bottom). **D.** T-type calcium current: the $_TCa^{2+}$ channel blocker NNC 55-0396 abolished a transient inward current (red, 10 µM), and abolished the single junction break potential in response to a current step in another cell (bottom). Current responses were recorded with the Cs^+ based intracellular solution in the presence of 20 µM TEA, and are averages of 40 responses. Voltage responses were recorded with the K^+-based intracellular solution.

Figure S3, related to Figure 2. Spike transfer attenuation from terminals to soma

A, B. Axonal filtering of typical Na^{+}- and Ca^{2+}-spike waveforms in a model of bipolar cell passive membrane properties. Axon length was 30, 50 or 70 µm, and axon diameters were 300 nm (blue), 200 nm (green) or 100 nm (red). The input spike waveforms are shown in black.

4. Supplementary References

1. Dreosti, E., Esposti, F., Baden, T., and Lagnado, L. (2011). In vivo evidence that retinal bipolar cells generate spikes modulated by light. Nature Neuroscience *14*, 951-952.
2. Burrone, J., and Lagnado, L. (1997). Electrical resonance and Ca^{2+} influx in the synaptic terminal of depolarizing bipolar cells from the goldfish retina. J Physiol *505 (Pt 3)*, 571-584.
3. Pan, Z.H., and Hu, H.J. (2000). Voltage-dependent Na(+) currents in mammalian retinal cone bipolar cells. J Neurophysiol *84*, 2564-2571.
4. Protti, D.A., Flores-Herr, N., and von Gersdorff, H. (2000). Light evokes Ca^{2+} spikes in the axon terminal of a retinal bipolar cell. Neuron *25*, 215-227.
5. Zenisek, D., Henry, D., Studholme, K., Yazulla, S., and Matthews, G. (2001). Voltage-dependent sodium channels are expressed in nonspiking retinal bipolar neurons. J Neurosci *21*, 4543-4550.
6. Palmer, M.J. (2006). Modulation of Ca(2+)-activated K^{+} currents and Ca(2+)-dependent action potentials by exocytosis in goldfish bipolar cell terminals. J Physiol *572*, 747-762.
7. Ohkuma, M., Kawai, F., Horiguchi, M., and Miyachi, E. (2007). Patch-clamp recording of human retinal photoreceptors and bipolar cells. Photochem Photobiol *83*, 317-322.
8. Cui, J., and Pan, Z.H. (2008). Two types of cone bipolar cells express voltage-gated Na^{+} channels in the rat retina. Vis Neurosci *25*, 635-645.
